# Sleep Disturbances and Non-REM Phase Alterations in Children with Celiac Disease: A Combined Questionnaire and EEG Study

**DOI:** 10.3390/brainsci16030304

**Published:** 2026-03-12

**Authors:** Mehpare Sarı Yanartaş, Nurel İnan Aydemir, Furkan Donbaloğlu, Chakan Tsakir, Özlem Yayıcı Köken, Burçin Şanlıdağ, Şenay Türe, Boran Şekeroğlu, Aygen Yılmaz, Şenay Haspolat

**Affiliations:** 1 Department of Pediatric Neurology, Faculty of Medicine, Akdeniz University, 07100 Antalya, Türkiye; 2 Antalya City Hospital, 07070 Antalya, Türkiye; 3 Department of Pediatric Neurology, Faculty of Medicine, Near East University, 99138 Nicosia, Cyprus; 4 Division of Social Pediatrics, Department of Pediatrics, Faculty of Medicine, Akdeniz University, 07100 Antalya, Türkiye; 5 Department of Computer Engineering, Faculty of AI and Informatics, Near East University, 99138 Nicosia, Cyprus; 6 Department of Pediatric Gastroenterology, Faculty of Medicine, Akdeniz University, 07100 Antalya, Türkiye

**Keywords:** celiac disease, sleep disturbances, sleep spindles, electroencephalography, pediatrics

## Abstract

**Highlights:**

**What are the main findings?**

- Sleep disturbances are frequently reported in children with celiac disease and appear to be more pronounced in those with poor adherence to a gluten-free diet.
- Lower sleep spindle amplitude and density are observed in children with higher sleep disturbance scores, suggesting an association between subjective symptoms and N2 sleep microarchitecture.

**What are the implications of the main findings?**

- These findings underscore the potential relevance of dietary adherence when interpreting sleep-related complaints in pediatric celiac disease.
- Sleep spindle metrics may represent promising objective correlates of symptom burden, warranting further validation in longitudinal and larger-scale studies.

**Abstract:**

**Background:** Celiac disease (CD) is a multisystem immune-mediated disorder increasingly recognized to affect sleep and neurobehavioral functioning. Pediatric data remain limited, and no prior study has examined especially for sleep microstructure in this population. This study evaluates the prevalence and patterns of sleep disturbances in children with CD using the Sleep Disturbance Scale for Children (SDSC) and explores potential electrophysiological correlates through N2 sleep spindle analysis. **Methods:** Children with biopsy-confirmed CD (*n* = 31) and age-matched controls (*n* = 25) completed the SDSC. A subgroup of CD patients with SDSC ≥ 35 and healthy controls underwent quantitative sleep spindle analysis (C3, C4, O1, O2) using automated and visual verification methods combined. **Results:** Clinically significant sleep disturbances were substantially more prevalent in CD than in controls (77.4% vs. 12%). Excessive somnolence, sleep–wake transition disorders, and sleep hyperhidrosis were the most affected domains. Moreover, among children with CD, those noncompliant with a gluten-free diet exhibited higher rates of excessive somnolence and sleep–wake transition disorders. While spindle parameters did not differ between groups, higher SDSC scores (≥35)—particularly in the somnolence and sleep–wake transition disorder domains—are associated with reduced spindle amplitude and density, suggesting that spindle alterations are linked to sleep disturbance severity rather than disease status per se. **Conclusions:** Sleep disturbances are common in pediatric CD and worsen with poor dietary adherence. Although sleep microarchitecture is largely preserved, reduced spindle activity is evident in children with higher subjective sleep burden, suggesting that spindle metrics may serve as potential objective markers for sleep disturbance. Longitudinal studies are required for validation.

## 1. Introduction

Celiac disease (CD) is a systemic immune-mediated disorder in which chronic inflammation, nutrient malabsorption, and neuroimmune mechanisms may contribute to a broad spectrum of neuropsychiatric manifestations, including irritability, attentional difficulties, and daytime fatigue [1,2,3]. Sleep disturbances in CD have been increasingly recognized and are thought to arise from immune dysregulation, elevated pro-inflammatory cytokines (e.g., TNF-α, IFN-γ, IL-6), and potentially anti-neuronal antibodies [3]. Identifying sleep-related problems in CD is clinically relevant because sleep plays a fundamental role in cognitive and emotional development as well as immune regulation [4]. Children with CD have been reported to exhibit sleep abnormalities using both subjective questionnaires and objective polysomnography (PSG), including increased sleep fragmentation, daytime sleepiness, and sleep-disordered breathing (SDB) [5,6,7]. However, recent PSG studies in newly diagnosed pediatric CD have primarily emphasized macrostructural sleep parameters (e.g., sleep stage distribution and fragmentation) [8], leaving key aspects of sleep microarchitecture insufficiently characterized [6].

Sleep microarchitecture—particularly sleep spindles—plays a critical role in pediatric neurodevelopment by supporting memory consolidation, executive function, and emotional regulation [9]. Sleep spindles are brief thalamocortical oscillations that are characteristic elements of N2 sleep and serve as sensitive markers of thalamocortical circuit integrity. They are generated by GABAergic neurons within the thalamic reticular nucleus (TRN) and expressed through distributed thalamocortical projections [10]. Although spindles are typically maximal over central derivations, their propagation and regional expression depend on network connectivity and may reflect region-specific vulnerabilities.

In pediatric CD, electrophysiological studies have documented occipital-predominant epileptiform abnormalities that correlate with tissue transglutaminase (tTG) antibody levels and may improve with a gluten-free diet (GFD) [11,12,13]. Such preferential regional involvement raises the possibility that immune-mediated disruption might cause sleep spindles over that specific region to be affected. Nevertheless, objective spindle-specific metrics (e.g., density, amplitude, frequency, and their modulation across the night) remain largely absent in the pediatric CD literature.

A mechanistic gap also persists regarding how systemic inflammation in CD may influence the neurophysiological generators of sleep. In broader neuroimmunological contexts, elevated pro-inflammatory cytokines have been associated with altered spindle characteristics and cognitive dysfunction in chronic inflammatory states [14]. Yet direct evidence linking cytokine-mediated inflammation to TRN excitability or spindle initiation in CD is lacking. Furthermore, although executive and behavioral regulation difficulties are reported in pediatric CD, their relationship to sleep spindle microarchitecture and sleep cycle organization (including NREM/REM cycling dynamics) has not been systematically explored [15,16].

Adherence to a GFD is increasingly recognized as beneficial not only for gastrointestinal pathology but also for neurological status and brain bioelectrical activity [13]. A recent systematic review reported improvement in several neurological and neuropsychiatric conditions—including epilepsy, attention-deficit/hyperactivity disorder, restless leg syndrome, and developmental delay—following GFD initiation, with multiple studies demonstrating statistically significant benefits [17].

Therefore, the present study aims to evaluate the prevalence and characteristics of sleep disturbances in children with CD evaluated by the Sleep Disturbance Scale for Children (SDSC) and to investigate electrophysiological correlates by quantifying N2 sleep spindle dynamics. Specifically, we quantified spindle density, amplitude, and frequency over central and occipital regions to assess whether alterations in spindle architecture may serve as objective neurophysiological markers of sleep disturbance in pediatric CD. We hypothesized that children with CD would exhibit a higher prevalence of clinically significant sleep disturbances than healthy controls and sleep spindle metrics might be used as an objective evaluation parameter for sleep disturbances.

## 2. Materials and Methods

### 2.1. Ethical Approval

Ethics approval was obtained from the Clinical Studies Ethical Committee of Akdeniz University (KAEK-824). Written informed consent was obtained from all parents/legal guardians. The study adhered to the STROBE guidelines. The study was conducted in accordance with the Declaration of Helsinki.

### 2.2. Recruitment and Study Design

This prospective cross-sectional study included children aged 4–18 years who had been diagnosed with CD at the Akdeniz University Pediatric Gastroenterology and Neurology Division between November 2023 and December 2024. Eligible participants who were diagnosed with CD between 2019 and 2023 were identified retrospectively from hospital records; however, all study-related outcome assessments, including SDSC administration and EEG analyses, were conducted prospectively after ethics approval was obtained in 2023. Healthy controls were recruited from children evaluated in the pediatric neurology clinic for benign conditions (idiopathic tremor or isolated fine motor delay with normal neuro exam and normal imaging, no DSM diagnosis). Given the lack of definitive prevalence data on sleep disturbances in the general pediatric and adolescent population, and the absence of evidence indicating an increased prevalence in the selected benign neurological cohort compared with the general population, this patient group was considered appropriate as a control group that had no gastrointestinal, neurological, or psychiatric disorders.

Exclusion criteria were:

• any psychiatric diagnosis;
• use of psychotropic medication;
• neurological disease other than CD-related symptoms;
• chronic pulmonary disease/adenotonsillar hypertrophy requiring surgery/known OSA diagnosis;
• regular use of medications affecting sleep (antihistamines, melatonin, corticosteroids, stimulants);
• iron deficiency anemia/restless legs syndrome.

Demographic and clinical data were retrieved from medical records. These included age, sex, age at diagnosis, duration of follow-up, dietary compliance, and comorbidities such as type 1 diabetes mellitus (T1D) and Hashimoto thyroiditis.

Adherence to a GFD was evaluated through structured medical interviews. Assessment included caregiver-reported dietary history, presence of gastrointestinal symptoms (e.g., abdominal pain, bloating), and growth and developmental parameters. Although follow-up serological monitoring was not uniformly available, dietary compliance was assessed using this multidimensional clinical approach.

Because T1D may independently influence sleep and was unevenly distributed between dietary-compliant and noncompliant participants, analyses were stratified by T1D status (yes/no).

### 2.3. Sleep Disturbance Assessment

Sleep disturbances were evaluated using the SDSC, administered to the primary caregiver by the same research team (M.S.Y., F.D., C.T.). The SDSC consists of 27 Likert-type items validated in Turkish [18,19] and assesses six domains:

1. disorders of initiating and maintaining sleep;
2. sleep breathing disorders;
3. disorders of arousal;
4. sleep–wake transition disorders;
5. excessive somnolence;
6. sleep hyperhidrosis.

A total score ≥35 indicates clinically significant sleep disturbance.

### 2.4. EEG Subgroup and Recording Protocol

Fourteen children with CD (SDSC ≥ 35) and 13 age- and sex-matched healthy controls were included in the spindle analysis. EEG recordings for both CD participants and controls were performed under identical recording conditions. All participants underwent standard video-EEG recordings using the international 10–20 system during spontaneous daytime sleep.

To promote sleep onset, both CD participants and controls were partially sleep-deprived and instructed to be awake from 03:00 a.m. on for the recording day. EEG sessions were conducted between 10:00 a.m. and 12:00 p.m. No sedation was used. Recordings continued until spontaneous sleep occurred, and at least 20 min of consolidated N2 sleep was required for inclusion in the spindle analysis. If insufficient sleep was obtained, repeat appointments were scheduled.

Sleep staging was independently confirmed by two experienced neurophysiologists who were blinded to group allocation.

Technical parameters:

• sampling rate: 500 Hz;
• bandpass filter: 0.5–40 Hz;
• Analyses focused on C3, C4, O1 and O2 electrodes.

Sleep spindle topography shows predominant frontocentral and centroparietal distribution reflecting thalamocortical network organization; therefore, spindle analyses were primarily conducted over central derivations [20,21]. Occipital electrodes were additionally reviewed due to reported occipital EEG abnormalities and increased occipital epileptiform activity in pediatric CD [11].

### 2.5. EEG Signal Processing

EEG recordings were archived and analyzed at the Pediatric EEG Laboratory of Akdeniz University and Near East University. All participants underwent a single sleep EEG recording using a Nihon Kohden system (Shinjuku-ku, Tokyo, Japan) with 18 channels. Scalp electrodes were positioned according to the international 10–20 system. Recordings consisted of a 30 min digital tracing, including at least 20 min of consolidated N2 sleep.

EEG preprocessing and signal analysis were conducted using the MNE (Minimum-Norm Estimation)-Python toolbox (version 1.5.1). Signals were bandpass-filtered between 0.5 and 40 Hz. For sleep staging, data were downsampled to 100 Hz. Sleep stages were automatically predicted using pretrained machine-learning algorithms implemented in the YASA (Yet Another Spindle Algorithm) toolbox (version 0.6.4) and were independently reviewed by two experienced neurophysiologists blinded to group allocation.

Sleep spindle detection was performed using YASA with default detection parameters (frequency range 11–16 Hz; duration 0.5–2 s; amplitude and power thresholds relative to background sigma activity). Primary spindle analyses were conducted over central derivations (C3 and C4), given the known frontocentral predominance of spindle activity reflecting thalamocortical network organization. Occipital derivations (O1 and O2) were additionally analyzed as secondary exploratory regions due to previously reported occipital EEG abnormalities in pediatric celiac disease.

All automatically detected spindle events were independently visually reviewed by two blinded pediatric neurophysiologists, who accepted or rejected events based on standard morphological criteria (waxing–waning sigma rhythm, absence of artifact contamination). Discrepancies were resolved by consensus.

### 2.6. Statistical Analysis

Statistical analyses were performed using SPSS version 22.0 (IBM Corp., Chicago, IL, USA). Normality of continuous variables was assessed using the Kolmogorov–Smirnov test, and homogeneity of variances was evaluated with Levene’s test. Continuous variables were expressed as mean ± standard deviation (SD) for normally distributed data and as median (min–max) for non-normally distributed data. Categorical variables were presented as frequencies and percentages. Between-group comparisons were performed using Student’s *t*-test for continuous variables with normal distribution and the Mann–Whitney U test for non-normally distributed data. Categorical variables were compared using Pearson’s chi-square test or Fisher’s exact test, as appropriate. Correlations were assessed using Pearson or Spearman coefficients, depending on distribution. Effect sizes were reported as Cohen’s d for independent samples *t*-tests and as r for Mann–Whitney U tests, where applicable. Given the exploratory nature of spindle and subscale analyses, no formal correction for multiple comparisons was applied. A two-tailed *p*-value <0.05 was considered statistically significant. Within each stratum, SDSC total and subscale scores were compared between compliant and noncompliant participants (Student’s *t*-test or Mann–Whitney U test, depending on distribution). A sensitivity analysis excluding participants with T1D was also performed.

## 3. Results

A total of 56 children were included in the study: 31 with CD and 25 healthy controls. Gender distribution was similar between groups (male: 51.6% vs. 52%), and mean age at evaluation did not differ significantly (114.2 vs. 118.8 months). Among patients with CD, the mean age at diagnosis was 84.6 months. Comorbidities included T1D in 29% and Hashimoto thyroiditis in 6.5% of patients.

### 3.1. Sleep Disturbance Profiles (SDSC Scores)

Children with CD demonstrated significantly higher SDSC total scores (40) compared with controls (28), *p* < 0.001. Clinically significant sleep disturbance (SDSC ≥ 35) was present in 77.4% of children with CD, while only 12% of controls exhibited such disturbances (*p* < 0.001).

Subdomain analysis showed that the CD group had significantly higher prevalence of excessive somnolence (8 participants in the CD group vs. 5 in the comparison group, *p* = 0.034), sleep hyperhidrosis (4 vs. 2, *p* = 0.045), and sleep–wake transition disorders (6 vs. 3, *p* = 0.023). There were no significant differences between groups for disorders of arousal (*p* = 0.956), disorders of initiating and maintaining sleep (*p* = 0.350), or sleep breathing disorders (*p* = 0.583) (Table 1).

### 3.2. Sleep Disturbance According to Dietary Adherence

Among the 31 children with CD, dietary compliance data were available for 29 participants (two excluded due to missing compliance information). Seventeen children were classified as noncompliant and twelve as compliant with GFD. The prevalence of T1D was higher in the noncompliant group (41.2% vs. 16.6%; Fisher’s exact test, *p* = 0.034).

Excessive somnolence (*p* < 0.001), sleep–wake transition disorders (*p* < 0.001), and total SDSC scores (*p* < 0.001) were higher in the noncompliant group. However, the proportion exceeding the clinical cutoff (SDSC > 35) did not differ significantly between groups (*p* = 0.074) (Table 2).

Higher scores in excessive somnolence (*p* < 0.001) and sleep–wake transition disorders (*p* < 0.001) were particularly notable among noncompliant children. T1D was more prevalent in the noncompliant group (41.2% vs. 16.6%, *p* < 0.001). Gender distribution and most other SDSC subdomains did not differ significantly (Table 2).

### 3.3. Sleep Spindle Analysis in EEG Recordings

Sleep spindle parameters were evaluated in 27 participants (14 CD patients with SDSC ≥ 35 and 13 controls). There were no significant differences between groups in spindle count, frequency, amplitude, or density at C3, C4, O1, or O2 derivations (all *p* > 0.05). Hemispheric comparisons (C3 vs. C4, O1 vs. O2) also showed no asymmetry in either group.

### 3.4. Relationship Between Sleep Disturbances and Spindle Features

Within the CD subgroup, sleep spindle characteristics were examined according to SDSC subscale profiles. Children with higher scores in excessive somnolence and sleep–wake transition disorders demonstrated significantly reduced spindle amplitude and spindle density (*p* < 0.05 for both), whereas other subdomains showed no significant associations (Table 3). Correlation analysis revealed that higher total SDSC scores correlated with lower spindle amplitude and density.

## 4. Discussion

Sleep plays a fundamental role in children’s cognitive, emotional, and physical well-being, and sleep disruption may impose a disproportionate burden in chronic medical conditions [22]. In this study, clinically significant sleep disturbance was substantially more frequent in children with CD than in healthy peers (77.4% vs. 12%). This finding is consistent with broader evidence that sleep problems contribute to disease burden and reduced quality of life in chronic pediatric disorders [3,23], and supports routine screening for sleep-related symptoms during follow-up of children with CD.

Although research on sleep in pediatric CD remains limited, available studies are broadly concordant with the present results. Reiter et al. reported higher SDSC scores in children with CD compared with healthy controls, with sleep complaints also present in children with unexplained abdominal pain [6]. Similar patterns have been described in adult cohorts using the Pittsburgh Sleep Quality Index (PSQI) [24]. Together, these findings suggest that sleep disturbances may represent a clinically relevant, cross-age feature of CD and merit systematic assessment.

For the SDSC subdomain analysis, children with CD experienced excessive somnolence, sleep hyperhidrosis and sleep–wake transition disorders significantly more when compared to the control group.

Among SDSC subdomains, excessive somnolence showed the strongest association with CD and its prevalence was also higher in CD patients who were nonadherent to a gluten-free diet (GFD) compared to the compliant group of patients. Daytime sleepiness and fatigue—often reported as burdensome extraintestinal symptoms—may relate to inflammatory activity, nutrient malabsorption, or both, and can contribute to “brain fog,” attentional difficulties, and impaired quality of life [25,26,27]. Findings of this study underscore hypersomnolence as an important and potentially underrecognized feature in pediatric CD. Sleep hyperhidrosis was also more frequent in CD, a finding consistent with observations of altered autonomic or thermoregulatory regulation in sleep-related conditions, although its mechanisms in CD remain unclear [28]. In contrast, no group differences in disorders of arousal, and sleep-disordered breathing scores were observed between groups. While some studies have proposed increased OSA risk in CD, pediatric findings remain unclear [1,12] and cannot be supported with this study.

The sleep–wake transition subscale was significantly elevated in both the CD vs. control group of patients and in noncompliant vs. compliant CD groups of patients. Transitions between wakefulness and sleep depend on coordinated neuromodulatory and cortical network shifts [29]; this pattern may reflect reduced arousal stability in CD, particularly in the setting of persistent gluten exposure or higher symptom burden.

Nonadherence to a GFD was associated with higher overall SDSC scores in this cohort, suggesting a relationship between dietary adherence and sleep-related symptoms. Prior literature, however, is unclear. Some studies report improvement of sleep-related symptoms after GFD and others report persistent sleep difficulties despite adequate dietary control [3,24]. Importantly, the higher prevalence of type 1 diabetes mellitus (T1D) in the nonadherent group represents a meaningful potential confounder, as T1D is independently associated with sleep disturbance, including excessive daytime sleepiness and sleep fragmentation related to nocturnal glycemic variability [30,31]. Accordingly, the association between dietary nonadherence and sleep disturbance should be interpreted cautiously and cannot be taken as evidence of a direct causal effect of gluten exposure alone. Larger studies with multivariable modeling and objective measures of dietary adherence and inflammatory activity are required to clarify those independent contributors.

To date, EEG-based studies in CD have primarily focused on epileptiform abnormalities [2,32]. No prior report addressing sleep spindle parameters in non-REM sleep in CD has been documented within the English literature. Although nocturnal sleep offers a longer recording period and thus a greater opportunity for comprehensive spindle sampling, converging evidence suggests that spindle characteristics demonstrate strong trait-like stability. Daytime naps reliably capture core spindle metrics—including density, frequency, and duration—in both adults [33] and infants [34], and predict memory consolidation outcomes across age groups [34,35]. The spectral profile and topographical distribution of non-REM sigma activity remain highly consistent within individuals across nights and experimental conditions, supporting the concept of spindle activity as an electrophysiological “fingerprint” [36,37]. Therefore, daytime nap EEG recordings were employed in this group of patients to enable the assessment of spindle features within a feasible and developmentally appropriate recording framework. In the present study, conventional spindle metrics (count, frequency, amplitude, density), recorded during daytime sleep, did not differ between CD patients and healthy controls, and no hemispheric asymmetry was identified. These findings suggest preserved thalamocortical function at the level of basic sleep physiology.

Notably, within the subgroup of CD participants with clinically significant sleep disturbance (SDSC ≥ 35), higher excessive somnolence and sleep–wake transition scores were associated with reduced spindle amplitude and density. This pattern aligns with emerging evidence that subjective sleep disturbance severity may relate to measurable changes in spindle dynamics. Rather than functioning as a disease-specific discriminator, spindle metrics in this cohort appeared to index symptom burden among affected children.

Strengths of this study include the combined assessment of sleep using a validated questionnaire and objective EEG-based microarchitecture metrics. Limitations include the cross-sectional design, the absence of baseline pre-diet measures, non-uniform availability of serological follow-up for dietary adherence assessment, and the relatively small EEG subgroup. In addition, recordings were obtained during daytime sleep rather than consolidated nocturnal sleep; therefore, generalization to habitual nighttime sleep should be made cautiously. Future prospective, multicenter studies incorporating full-night recordings, objective markers of GFD adherence and inflammation, and neurocognitive outcomes are warranted to clarify mechanisms and clinical implications.

## 5. Conclusions

Sleep disturbances are markedly more prevalent in children with CD, particularly excessive somnolence and sleep–wake transition difficulties. While spindle metrics did not differ between CD and controls at the group level, greater subjective sleep disturbance—especially excessive somnolence and sleep–wake transition disorder—is associated with reduced N2 spindle density and amplitude within the CD subgroup. These findings suggest that spindle features may reflect symptom severity rather than a disease-specific marker. Routine screening for sleep problems in pediatric CD may help identify at-risk children and guide timely interventions. Additionally longitudinal studies are warranted to validate spindle metrics as potential objective biomarkers.

## Figures and Tables

**Table 1:** Summary of the general SDSC values of patients with CD and healthy control.

	Celiac Disease (*n*:31)	Healthy Controls (*n*:25)	*p*-Value
**Gender (male %, ** ***n*** **)**	51.6 (16)	52 (13)	-
**Mean age at study (months, mean, min–max)**	114.2 (58–216)	118.8 (70–207)	-
**Mean age at diagnosis (mean, min–max)**	84.6 (22–156)	-	-
**T1D as a comorbidity (%, ** ***n*** **)**	29 (9)	-	-
**Hashimoto thyroiditis as a comorbidity (%, ** ***n*** **)**	6.5 (2)	-	-
**The score of disorders of arousal (mean, min–max)**	4 (3–7)	3 (3–7)	0.956
**The score of disorders of initiating and maintaining sleep (mean, min–max)**	11 (8–22)	9 (7–15)	0.350
**The score of excessive somnolence (mean, min–max)**	8 (5–16)	5 (5–13)	**0.034**
**The score of sleep breathing disorders (mean, min–max)**	4 (3–8)	4 (3–7)	0.583
**The score of sleep hyperhidrosis (mean, min–max)**	4 (2–9)	2 (1–4)	**0.045**
**The score of sleep–wake transition disorders (mean, min–max)**	6 (4–15)	3 (4–8)	**0.023**
**Total score of SDSC (mean, min–max)**	40 (27–62)	28 (24–40)	**<0.001**
**Total score of SDSC > 35 (%, ** ***n*** **)**	77.4 (24)	12 (3)	**<0.001**

Bold values indicate statistically significant results (*p* < 0.05).

**Table 2:** Summary of the SDSC values of children diagnosed with CD according to GFD compliance. *(Two patients were excluded due to missing compliance data).*

	Noncompliant Group (*n*:17)	Patients with GFD (*n*:12)	*p*-Value
**Gender (male, %, ** ***n*** **)**	52.9 (9)	58.3 (7)	0.581
**Mean age at study (months, mean, min–max)**	128.9 (58–216)	123.5 (71–176)	**0.047**
**Mean age at diagnosis (mean, min–max)**	89.2 (22–156)	92.2 (28–150)	**0.028**
**T1D as a comorbidity (%, ** ***n*** **)**	41.2 (7)	16.6 (2)	**<0.034**
**Hashimoto thyroiditis as a comorbidity (%, ** ***n*** **)**	11.8 (2)	-	-
**The score of disorders of arousal (mean, min–max)**	5 (3–7)	4 (3–7)	0.657
**The score of disorders of initiating and maintaining sleep (mean, min–max)**	14 (8–20)	14 (9–22)	0.565
**The score of excessive somnolence (mean, min–max)**	10 (5–16)	8 (5–11)	**<0.001**
**The score of sleep breathing disorders (mean, min–max)**	5 (3–8)	5 (3–8)	0.456
**The score of sleep hyperhidrosis (mean, min–max)**	4 (2–9)	4 (2–8)	0.512
**The score of sleep–wake transition disorders (mean, min–max)**	8 (4–15)	6 (4–11)	**<0.001**
**Total score of SDSC (mean, min–max)**	47 (27–62)	40 (29–50)	**<0.001**
**Total score of SDSC > 35 (%, ** ***n*** **)**	88.2 (15)	83.3 (10)	**0.074**

Bold values indicate statistically significant results (*p* < 0.05).

**Table 3:** Comparison of sleep spindle features across SDSC subscales in CD patients with sleep disturbance (*n*:14).

SDSC Subscale	Mean Spindle Amplitude (µV)	Mean Spindle Density (Spindles/min)	*p*-Value (Amplitude)	*p*-Value (Density)
**Disorders of Arousal**	37.5	3.2	0.412	0.374
**Disorders of Initiating and Maintaining Sleep**	36.8	3.0	0.381	0.417
**Excessive Somnolence**	31.2	2.2	* 0.022	* 0.017
**Sleep Breathing Disorders**	35.6	2.9	0.338	0.390
**Sleep Hyperhidrosis**	34.1	2.5	0.299	0.266
**Sleep–Wake Transition Disorders**	32.7	2.3	* 0.031	* 0.024

* *p* < 0.05 was considered statistically significant. Sleep spindle amplitude and density showed the most marked reduction in CD patients with high scores in excessive somnolence and sleep–wake transition disorders subscales.

## Data Availability

The data presented in this study are available on request from the corresponding author due to privacy and ethical reasons.
